# Prenatal care and human rights: Addressing the gap between medical and legal frameworks and the experience of women in Brazil

**DOI:** 10.1371/journal.pone.0281581

**Published:** 2023-02-14

**Authors:** Camila Brito Rodrigues, Erika Barbara Abreu Fonseca Thomaz, Rosângela Fernandes Lucena Batista, Pía Riggirozzi, Dina Stefany de Oliveira Moreira, Laura Lamas Martins Gonçalves, Zeni Carvalho Lamy

**Affiliations:** 1 Federal University of Maranhão, São Luís, Maranhão, Brazil; 2 Department of Public Health and Postgraduate Program in Public Health, Federal University of Maranhão, São Luís, Maranhão, Brazil; 3 Department of Politics and International Relations, University of Southampton, Southampton, United Kingdom; 4 Psychology Course, University of Vale do Rio dos Sinos, Porto Alegre, Rio Grande do Sul, Brazil; Indian Institute of Dalit Studies (IIDS), INDIA

## Abstract

Access to quality and affordable healthcare is central to the fulfilment of women’s reproductive and sexual health needs and rights. For this reason, the World Health Organization declared access to appropriate healthcare services during pregnancy and childbirth a fundamental women’s right. Prenatal care is a recognized human right to women’s health in Brazil, as declared by the 1988 Constitution and many Brazilian policies. However, implementing the rights to health in Brazil presents a fundamental performance gap between legal rights and their delivery concerning reproductive health. Through extensive fieldwork including focus groups, interviews with women and participate observation in two municipalities in northeastern Brazil, this article addresses these issues and explores women’s lived experience of access to and their fulfilment of the right to health regarding prenatal healthcare. We offer and account of the experience of women regarding what they identified as barriers that trample their right to health, that is: a) limited personnel and medical equipment as a perception of neglect; b) timely delivery of services: time matters for perception and experience of rights; c) misinformation as a barrier to the exercise of health rights; and d) socioeconomic barriers. These barriers particularly affect the right of women in rural communities, with lower socioeconomic levels and education, as well as brown and black women, from an intersectionality perspective, who are already at greater health risk and inadequate prenatal care. As such, we argue there is a performance gap between what the normative and legal frameworks encourage the health system to do and what the system actually provides in terms of access, equality, respect and continuity of treatment amongst certain groups in society whose right to health are denied while their health risks increase.

## Introduction

Prenatal care is an essential part of Primary Healthcare (PHC) affecting the health of women, pregnancy and childbirth, as such it is a central component of the right to health of women and child. For this reason, prenatal care has been recognized by the World Health Organization (WHO) as central to the reduction of maternal and perinatal mortality and key to reaching the United Nations Sustainable Development Goals [1].

In Brazil, prenatal care is a recognized human right to women’s health, as declared in its 1988 Constitution and in the Rede Cegonha Ordinance which establishes indicators of quality of care [2]. However, in practice, the provision of the right to prenatal care is uneven and not fully guaranteed across society, affecting, in particular, the rights and health of women in rural areas in the Northeast area of the country. This is problematic in a country with a maternal mortality rate of 60 deaths/100,000 live births (estimate for the year 2017) and where only half of the women in the Northeastern region have received adequate prenatal care [3, 4]. Inadequate prenatal care also leads to high rates of infant mortality in the country due to prematurity, low birth weight, and perinatal infection [5].

Many difficulties account for the limitations women experience in accessing quality prenatal care; including women’s lack of knowledge about their rights which not only acts as barrier to access but also as barrier to the claim of the right to health [6]. As such, enhancing opportunities to access and the enjoyment of the right to health goes hand in hand with the enhancement of the knowledge of rights, all of which has the potential to empower women who can act upon that knowledge [7, 8].

Given this context, the PHC has a fundamental role as a gateway to the health system and provider of the right to healthcare. PHC can strengthen actions aimed at women and family and community, guaranteeing resolutive access to services and respecting the cultural, sexual, ethnic, and religious diversity, contributing to the construction of women’s autonomy [9]. For the fulfilment of such a role, the territorialization [10] of PHC has been recognized as an important social and political process supporting the implementation of the constitutional principles of the right to health. Territorialization is a model or healthcare delivery based on the guidelines of the Brazilian Unified Health System (SUS) based on a territorial base delimiting a registered area for the delivery of services. Its fundamental principle is the understanding the values, practices, and vulnerabilities of the population. Based on that a health team organizes its work based on mapping individuals and/or families exposed to risks and vulnerabilities in their activity area [10]. This knowledge is essential, especially in rural communities, with lower socioeconomic levels and education and specific ethnic and racial groups, such as brown and black women, that are at greater risk of marginalization and of receiving inadequate prenatal care [11, 12].

Having PHC as a reference in the territory is central to the care and access to basic rights of specific populations, such as pregnant women and *quilombola* communities, an ethnic-racial group directly descendant of African slaves who settled in communities, which are often rural areas [13]. However, the enjoyment of rights in this type of communities will depend on qualified and cultural-sensitive provision of care, health education actions, and pro-active promotion of safe pregnancy and pre/postnatal care [2, 9]. These actions are embedded in rights, such as the right to have prenatal consultations with professionals from Medicine, Nursing, and Dentistry; access to exams and vaccination; adequate infrastructure and transport that allow access to Basic Health Units (BHU) and other health services; to link the place where the birth will take place and the elaboration of the delivery plan, in addition to social and labor rights [2].

The guarantee of these rights can help reduce maternal and child mortality, which in Brazil still hits hard due to preventable causes, including inadequate assistance in many places [14]. Despite having shown an essential reduction in recent years, maternal and child mortality in Brazil remains high [3].

This is a context far too common not only in Brazil but across developing countries where fundamental performance gap manifest between what the legal and normative frameworks tell states and health systems to do and how the delivery of reproductive health is in practice [15, 16]. This article addresses these issues by exploring women’s perspectives, knowledge and experiences of prenatal healthcare services. We argue that access, equality in treatment, respect and information about treatment tramples human rights principles and women’s experiences of prenatal healthcare delivery. We explore this argument with a focus on antenatal healthcare in two municipalities in Maranhão, one of the poorest states in the Northeastern region in Brazil.

By exploring the lived experience of women from urban and rural, as well as ethnic communities in Maranhão, we uncover barriers to the fulfilment of health needs and rights while contributing to debates about quality of prenatal care and the academic debate.

## Methods

This work is part of a funded project entitled “Engaging Users for Quality Enhancement and Rights: Strengthening the maternal and child healthcare system over the first 1,000 days in Brazil (EU QUERO)”. The research observed fully the appropriate ethical guidelines set out for research in Brazil and by the Universities of Southampton and approved by the Ethics Committee, CAAE number 92281818.9.1001.5086. All participants signed the written informed consent. The COREQ checklist was used as a guideline for writing the article.

This is a qualitative, descriptive, and analytical study carried out through in-depth fieldwork involving interviews and focus groups with women identified by key informants in the health system. Two municipalities in the state of Maranhão were included, one urban and one rural, referred to as municipalities A and B, respectively. Maranhão is one of the states with the worst human development index (HDI) in Brazil, located in the Northeast of the country, with high rates of maternal and child mortality. Data collection took place from January to May 2019. These municipalities were chosen because they belong to a health region with the Regional Action Plan of the Rede Cegonha. Also, they have significant socioeconomic and cultural differences.

Municipality A is the most important city of the state, and it is divided in nine health districts with large hospitals and maternity units. This is an urban municipality with medium HDI (from 0.5 to 0.799), and more than one million inhabitants [17, 18]. The research was carried out in the most urbanized health district covering all four existing BHU.

Municipality B is rural, with a low HDI (below 0.499) and less than 22 thousand inhabitants [17, 18]. The majority of the population is brown/black, poor, and predominantly quilombola. There are around 200 communities in the municipality, 90% of which are remnants of quilombos, characterized by black ancestry descending from slaves and keep specific territorial relations with their own cultural practices and historical trajectory [19]. There are nine BHU in this municipality, and all were included in the study.

The inclusion criteria for the selection of participants in this study were being a pregnant woman or mother with children up to two years of age who used one of the PHC facilities in municipalities A and B. Among these women, the selection sought to contemplate a diversified group according to marital status, geographical distance from home to BHU, age, number of children. The sample was intentionally selected by the community health workers (CHW). The saturation criterion was used to close the sample.

The technique used was the focus group (FG) type collective interview, a form of a group interview aiming to gather information about a specific topic [20]. All FG were conducted by an interdisciplinary team of female researchers from the fields of social and political sciences, and public health, from two Universities involved in this Project, the Federal University of Maranhão (Brazil) and the University of Southampton (United Kingdom). In addition, the work was supervised and coordinated by researchers, professors, with PhDs in the areas of social and political sciences as well as in the area of public health. The FG facilitators, with an extensive experience in this type of methodology and data collection technique, guided the debate, seeking to create a favorable environment for a natural and free conversation with each other. Each FG also had an observer who recorded the dynamics of the group, and a reporter who wrote down the sequence of the speeches to support the subsequent transcription. Interviews were not repeated, but two initial FG were conducted as pilots for team training and standardization, as well as to identify difficulties and possible sources of bias. These FG were not included in the sample.

Two data collection instruments were used: 1) structured questionnaire to obtain sociodemographic data and 2) script for the FG addressing questions about the participants’ perception of prenatal rights, how these rights are guaranteed, and what hinders and/or facilitates this guarantee. The script for the FG was validated (pilot tested) with a group of CHW and women.

The health units designated for the implementation of FG were agreed with the managers of the State and Municipal health departments. Subsequently, contacts were made with the managers of the BHU to schedule the first exploratory visit, seeking to get closer to the field and established a prior relationship to study commencement. With the support of the Family Health Strategy (FHS) team in each BHU, especially the CHW, women who met the inclusion criteria were identified and invited by telephone or face-to-face to the focus groups on previously agreed days and locations.

At point of contact with the participants and before the start of each FG, the purpose of the study as well as the dynamic of the FG was introduced to the women by the facilitator. A Consent and Participant Information form was provided and read aloud to all participants before signing. It was made it clear that participation was voluntary, that there were no right or wrong answers, that confidentiality was guaranteed, and that participants were free to express themselves and to tell their experiences in a respectful and honest manner. It was also confirmed that the results of the study would be used to prepare a manual of rights [21] as basis for further training of CHW in support of the goal of enhancing quality of healthcare services and provision of rights to women.

The FG were carried out in BHU in both municipalities, and in places close to the health service, such as churches and schools, bringing together participants from nearby units, in order to facilitate transport of the interviewees and ensure a comfortable environment for conducting the conversations. Three FG were carried out in municipality A and five in municipality B with an average duration of 1 hour and 30 minutes, recorded and later transcribed. Data were transcribed into text files and organized into excel spreadsheets, but no software was used for analysis. In addition, transcripts were not returned to participants for comment and/or correction, but two different recordings were made for each FG (and both were listened to for transcriptions) and two people independently made duplicate transcripts. A third researcher validated the transcripts.

The final number of women participating was 10 in municipality A and 46 in municipality B. The participation of women presented significant challenges because some were pregnant and/or mothers of young children, but there was no direct refusal. A lower number of FG and women in municipality A was due to: i) Inclusion of only one of the nine health districts in municipality A (corresponding to four BHU), while all districts in municipality B were included (nine BHU); ii) In municipality B, due to its peculiar characteristics (quilombola rural population), there was a greater diversity of situations of vulnerability experienced by women, requiring a larger sample size to cover your singularities; and iii) A smaller number of interviews was needed in municipality A to reach the saturation criterion for discontinuing data collection.

Data analysis was carried out using content analysis-thematic modality by the authors who read through the verbatim transcriptions of all audio-recordings and coding process [22]. After exhaustive reading of the interviews, data coding was carried out seeking to extract the relevant themes and subsequently group them into categories and then interpret the content, articulating the speeches with their production context [23]. Three main coders were used to organize and interpret the data: (i) notions and interpretations of rights to health as recognized by women; (ii) rights identified by women but not respected by the system as per participants’ accounts; (iii) barriers to the access and quality of prenatal care. From this coding, the results were categorized in the thematic cores.

To guarantee the participants’ anonymity, the code “A” was used for FG of municipality A and “B” for groups of municipality B, followed by the FG order number and fictitious names were adopted for women. The names of the maternity hospitals and municipalities mentioned by the women during the interviews were also replaced. Participants received feedback on the study results, and training on women’s and children’s rights in the first 1000 days of life was conducted in each municipality.

## Results and discussion

This section is divided in three parts. Part one explores the sociodemographic characteristics of interviewed women. Part two discusses the main barriers identified by these women. Part three presents the right pointed as the more consolidated: the vaccines. The characteristics of the women whose speeches were selected to illustrate the results of this study are shown in Table 1.

**Table 1:** Socio-economic and demographic data of the women. Maranhão, Brazil. 2019.

Woman	Sociodemographic characteristics
A1- Vanessa	Pregnant woman and mother of a child up to 2 years old, living close to the BHU, married, brown/black skin color, with 9 or more years of study, self-employed. The partner is the head of the family.
A1- Ana	Pregnant woman, married, black skin color, more than 9 years of study, self-employed (cleaner), whose partner is the head of the family, living near the BHU, in her own stilt house, with a family income of up to 1 MW. She is on her second planned pregnancy. Suffered 1 previous abortion–she has sickle cell anemia. She has no health insurance and has been receiving prenatal care since the 8th week of pregnancy. She had 1 prenatal consultation with a doctor and 4 with a nurse.
A1- Jessica	Pregnant woman, married, brown color, more than 9 years of study, self-employed, living near the BHU, with a family income up to 2 MW. The partner is the head of the family,
A2- Mariana	Pregnant woman, over 25 years old, married, brown/black color, 12 years of study, working, living close to the BHU, in a borrowed brick house, with family income between 1–2 MW, not receiving social benefits. The partner is the head of the Family. She has no history of abortion or dead child, and no health plan. Had access to prenatal care, starting at around 6 weeks, with 1 medical consultation and 4 with a nurse, but denies having received home visits from health professionals.
A2- Joana	Woman with a child up to 2 years old, 20–27 years old, in a consensual union, does not live near the BHU, white skin color, 9 years of study, unemployed, the partner is the head of the family, with a family income of up to 1 minimum wage, lives with several people in a rented brick house, receives social benefit, has no health insurance, had several prenatal consultations with a doctor and a nurse, starting in the first trimester, but no home visits by health professionals.
A3- Rosa	Pregnant woman, 20–29 years old, self-declared brown skin color, living in a consensual union, in a rented brick house close to the BHU, with a family income between 1 and 2 MW, a beneficiary of the Bolsa Família Program. She has access to a medical follow-up during prenatal care, starting in the first trimester.
B2- Cecilia	Pregnant woman and mother of a 2-year-old child, 26 years old, consensual union, brown skin color, housewife and sells bonbons, head of the family, lives far from the BHU, evangelical, less than nine years of study, up to 1 MW, lives with her partner and three children, lives in a borrowed brick house, 3 appointments with a doctor, 5 with a nurse.
B3- Alice	Postpartum woman, 33 years old, married, white skin color, teacher, head of the family, Catholic, more than 15 years of study, receiving 1 to 3 MW, living close to BHU, with 5 people (husband and children) in her own house of brick, not receiving social benefits. She had access to prenatal care in the public health system (4 consultations with a doctor and 2 with a nurse) and in the private network (2 consultations with a doctor).
B3- Luana	Pregnant woman, 32 years old, consensual union, brown skin color, Catholic, less than 8 years of education, housewife, head of the family, receives less than 1 MW, assisted by the Bolsa Familia Program, lives close to the BHU, with 6 people (partner and children), in a mud house. She did not have access to consultations with a doctor, but only 5 with a nurse.
B4- Lorena	Mother of a child up to 2 years old, 34 years old, brown skin color, 12 years of education, unemployed, but is the person with the highest income in the family (assisted by social benefit—Bolsa Escola, receiving less than 1 MW). She lives near the BHU, in her own brick house, with 7 people. Denies having a religion. She does not have health insurance, but she had access to prenatal care, starting in the 3rd month of pregnancy: she had 3 consultations with a nurse and received 3 home visits from CHW, but no consultation with a doctor.
B4- Julia	Pregnant woman, 18 years old, brown skin color, housewife, head of the family, no religion, consensual union, less than 12 years of study, up to 1 MW, assisted by the Bolsa Família Program, living far from BHU, with 3 people (partner and 2 children) in a borrowed mud house. She had only one single prenatal consultation (with a Nurse).
B4- Amelia	Mother of a 2-year-old child, 19 years old, single, brown skin color, housewife, head of the family, Catholic, 12 years of schooling, up to 1 MW, assisted by social benefit (Bolsa Família Program), living far from BHU, with 4 people (mother, sister, and daughters) in her own brick and mud house. Attended prenatal care: 3 consultations with a doctor, 4 with a nurse, and 1 home visit with a CHW.
B5- Ligia	Mother of a 2-year-old child, 25 years old, single, brown skin color, Catholic, less than 9 years of study, unemployed, farmer, receiving up to 1 MW, assisted by social benefits (Bolsa Família and Bolsa Escola Programs), living close to BHU, with 6 people in his own thatched house. Attended prenatal care: 5 appointments with a doctor and 7 with a nurse, and 3 CHW home visits.

BHU: Basic health unit. MW: minimum wages (MW was around U$ 240.00). CHW: Community health Workers.

### Part 1. Demography of interviewed women

In the eight FG carried out, 56 women were interviewed, aged from 15 to 35, 10 in municipality A and 46 in municipality B. Among the interviewees in municipality A, five were pregnant, one had an abortion and was trying to get pregnant, one puerperal woman and three mothers of children up to two years old (among them, one in a new pregnancy). All lived in urban areas and close to the BHU. Most reported being brown, married, having completed high school, and followed the Catholic or Evangelical religion. Most interviewees lived in brick houses with their partner and one to three other people in cohabitation. Most women were self-employed or homemakers, receiving social benefit resources, particularly Bolsa Família Program, a conditional cash transfer program launched in 2003 that focuses on increasing the number of clinical visits by pregnant women to the health service, child health, vaccination and education [24]. Most of the interviewees’ highest income comes from their partner, often not higher than the national minimum wage.

In municipality B, 22 pregnant women, two puerperal women, and 22 mothers of children up to two years of age (three of them in a new pregnancy) were interviewed. All women in the municipality live in quilombola territory. The majority lived far from their reference BHU, said they were brown skin color, were in a consensual union, had completed high school, and were Catholic. Half of the women lived in a brick house and the rest in a mud house. Most lived with their partner, children, and four to six cohabiting people. Most were unemployed, received Bolsa Família, and referred to monthly family income up to a minimum wage. Of the 46 interviewees, 17 reported being the person with the highest income in the family, 16 said another family member and 13 referred to the partner as having the highest family income.

Although there are similarities between the interviewees, women from municipality B are from rural territories, from quilombola communities, live farther from the BHU, and have more difficulty in accessing health services in general. To a large extent these characteristics define perceptions and experiences regarding prenatal care needs and rights, as well as opportunities and barriers to the access to healthcare and the fulfilment of the right to health by women in these communities. This became apparent in the FG discussions and interviews, all of which is explored in the next section.

### Part 2. The lived experience of barriers to the enjoyment of the right to health concerning access and quality of prenatal care

Women’s general understanding of human rights concerning access and quality of prenatal services highlighted four main barriers to the enjoyment of their rights: (a) limited personnel and medical equipment as a perception of neglect; (b) timely delivery of services: time matters for perception and experience of rights; (c) misinformation as a barrier to the exercise of health rights; and (d) socioeconomic barriers.

### Limited personnel and medical equipment as a perception of neglect

When asked about their rights during pregnancy, most women cited the right to prenatal consultations. All pregnant women interviewed reported that they were undergoing prenatal care. Of the puerperal women and/or mothers of children up to two years of age interviewed, only one did not perform the recommended minimum number of six consultations. Also, most interviewees started prenatal care in the first trimester, demonstrating good prenatal coverage for women participating in the study.

*“I think the greatest need*, *at first*, *would be*… *the follow-up*, *the right to have the assistance*.*”* [A3 –Rosa]

*“As far as I know*, *the government provides help through the unified system to have a follow-up with both the doctor and the nurse*.*"* [B2 –Cecilia]

In Brazil, prenatal care for pregnant women at usual risk is recommended by a team of professionals, including general practitioner, nurse, dentist, nursing assistant/technician and CHW. The total number of consultations must be at least six, with interspersed follow-up between doctor and nurse. Whenever possible, consultations should be carried out in accordance with the following schedule: until week 28, monthly; from the 28th to the 36th week, every two weeks; 36th to 41st week, weekly. Besides, the pregnant women should have at least one consultation with a dentist [25].

In the rural municipality, women cited the right to medical consultation and pointed out a weakness in the health system when referring that all prenatal care was performed only by the nursing healthcare professional. Women in municipality A did not mention this frailty.

*“There is always a great lack of doctors here; they never are*. *That’s why we usually have little consultation with the doctor and more with the nurse*. *Because she’s always here*, *constantly*, *from Monday to Friday*. *It’s something that’s our right*, *and we don’t have it*.*”* [B5 –Ligia]

*“Right now*, *there is no doctor*, *we are just followed-up by the nurse*. *And it’s difficult because in the pregnant woman’s booklet it says that we have the right to consult with the doctor and we don’t have it*.*”* [B4 –Lorena]

The report Birth in Brazil (2014) shows that half of the pregnant women in the North and Northeast regions had prenatal care performed by nurses, in contrast to the other regions of the country, where more than 90% of pregnant women were attended by doctors [26]. In Brazil, prenatal consultations with a doctor and nurse are complementary and not substitute. Physicians are responsible for evaluating and treating pregnant women, for early identification of risk signs, and referral to reference services required [25]. The lack of medical professionals is another negative aspect of prenatal care, which is often the reason for women not to have the minimum number of necessary consultations [27]. While in many countries, midwives perform roles of maternal and child care during pregnancy, childbirth and birth, in Brazil this role is taken by obstetric nurses [28]. However, shortage of such professionals limits provision while requiring greater investments for their expansion and training [29].

The interviewees did not mention the dental consultation during the prenatal period as a right and, when asked, only two women reported having performed it. This right was neither recognized nor guaranteed.

*“There’s a major lack of dentists in the countryside*.*”* [B4 –Amelia].

Compared to doctors and nurses, the lower coverage of oral health teams is a factor that makes it challenging to perform prenatal dental care [30]. Data from the National Program for Access and Quality Improvement in Primary Care (PMAQ-AB), which evaluated family health teams that had oral health teams, demonstrated a strengthening and qualification of dental care in prenatal care in the Northeast region in the period from 2011 to 2014 [31]. However, the coverage of oral health teams remains lower than the coverage of doctors and nurses in all Brazilian regions. Furthermore, this distribution is still uneven. Smaller-sized municipalities and women with lower incomes, such as those in our study, still have difficulty accessing this professional, especially because it is a rural area and difficult to access, with a predominance of populations remnants of quilombos, although the Brazilian policy for oral health understands the importance of guaranteeing dental services for this population [13, 31].

The lack of guidance, the low importance given to dental care, and cultural aspects may also explain the low adherence to prenatal dental care. The myth that pregnant women cannot undergo dental consultations is still very prevalent, mainly due to the fear that the procedures may affect pregnancy and this belief is often reinforced by health professionals themselves [32].

In a study conducted in a state in southeastern Brazil, the women interviewed pointed out that they were not informed about the need for dental care during pregnancy [33]. Considering that periodontal diseases during pregnancy increase the risk of pre-eclampsia, low birth weight, and prematurity, dental care, considered safe and effective, must be stimulated during pregnancy [32].

To reverse this low adherence to dental consultations during prenatal care, the Ministry of Health established dental consultations as a priority action, changing the PHC financing logic through the Previne Brasil program. Thus, the transfer of financial resources to the PHC, from the Union to the Municipalities, was linked to the fulfillment of goals initially agreed for seven indicators, one of which being dental consultations in prenatal care [34].

Having access to basic infrastructure for prenatal care was recognized as a right not guaranteed to all women interviewed.

*“There is also a lack of material because sometimes we want to hear the child and don’t have sonar*. *To measure the belly*… *sometimes it was lacking”* [A2 –Mariana]

It is also recommended that prenatal care be performed in a physical area with adequate conditions of hygiene, ventilation and privacy, with minimal equipment and instruments, such as gynecological examination table, sphygmomanometer, stethoscope, flexible measuring tape, doppler sonar, among others [25].

The PMAQ-AB, which assessed, among other issues, the adequacy of the infrastructure of the primary care network, demonstrated that prenatal care concerning the availability of infrastructure was considered inadequate in the country [35]. In the Northeast region, only 29% of health units were considered to have adequate infrastructure, being worse in the North and Center-West regions. This demonstrates that prenatal coverage in Brazil, even though widely distributed, is still carried out in inadequate conditions [35]. Such deficiencies can make it challenging to carry out consultations and exams, compromising the quality of prenatal care [30].

### Timely delivery of services: Time matters for perception and experience of rights

Most women from both municipalities recognized the performance of laboratory tests as a right. They reported that their access was facilitated by Rede Cegonha, reinforcing the importance of this strategy for the quality of prenatal care.

*“I participated in that Rede Cegonha and did everything for free*." [B4 –Lorena]

*“Rede Cegonha in the*… *laboratory*, *right*? *It is easier for us*.*"* [A2 –Joana]

According to Rede Cegonha Ordinance and other regulations, the pregnant woman has the right to perform prenatal laboratory tests and to access the results in a timely manner [2, 25]. However, for those in the rural municipality, the guarantee of this right was less effective, considering that they often had to travel to the capital or other municipalities or even seek the private service to receive the result promptly, which is especially serious, considering that they are poor women, who depend on income redistribution policies.

*“Pregnant women*, *sometimes*, *need a simple exam and have to move to another place*. *In our municipality*, *there is no support to do these exams*.*"* [B3 –Luana]

*“The exams that we are expected to have need to be done in the private sector*.*"* [B4 –Julia]

In addition to guaranteeing access to laboratory tests, the delivery of results promptly brings financial savings, safety and tranquility for pregnant women [36]. Although women from both municipalities reported the delay in the results, it is unclear whether women recognized the prompt delivery of results as a right.

Another exam recognized as a right by many interviewees was obstetric ultrasound. However, having this right guaranteed was reported as difficult in both municipalities.

*“We*, *at SUS*, *have a location that provides ultrasound*, *but for pregnant women*, *it’s much more difficult because it takes too long*! *So*, *we prefer to pay soon and receive it soon*.*”* [A1 –Vanessa]

This difficulty was reported in other studies, in which pregnant women needed to use the private service to perform the exam and receive the result promptly [37]. This reality needs to be modified since the performance of exams during prenatal care is of paramount importance, as it makes it possible to identify and treat comorbidities that already exist or that may occur during the pregnancy and come to affect the pregnant woman and her embryo [26].

### Misinformation as a barrier to the exercise of health rights

Regarding the right to receive information during the pregnancy-puerperal cycle, there were many reports that the guidelines offered by health professionals were insufficient, and women expressed the desire to receive more information about pregnancy, childbirth, and child care.

*“I think that during prenatal care*, *we had to have a class*, *get a baby or a doll*. *They should show*: *‘oh*, *when it is time to bathe*… *like this’*. *Like*, *about cleaning the navel*, *you know*? *Before it* [the umbilical cord] *falls*. *This*… *nothing is informed*. *We see it in the pregnant woman’s booklet*, *but not face to face*.*”* [A3 –Rosa]

*“It would be good if during the prenatal period the nurse*, *who is usually the one who makes the appointment*, *gave this support*, *thus*, *already prepared the psychological aspect for this part*, *for this part of the delivery*. *It usually doesn’t happen*, *you know*? *If you don’t search*, *don’t read for yourself*, *you don’t have it”* [B3 –Alice]

According to the Rede Cegonha Ordinance, it is women’s right and health professionals’ duty to implement social communication strategies and educational programs related to sexual health and reproductive health [2]. The woman and her family must receive information about prenatal care, hygiene care, physical activity, nutrition, bodily changes, fears, sexual activity, warning signs for childbirth, sexual and reproductive planning, care after childbirth with the women and newborns, mental health and domestic and sexual violence, legal benefits, among others [25].

In Brazil, the frequency of carrying out educational activities and guidance in prenatal care is still low. Developing educational strategies throughout prenatal care contributes to obtaining better obstetric results [38].

The women reported that prenatal consultations were restricted to observing laboratory tests. The information provided was often related to the recommendation to read the Pregnant Woman’s Booklet, with no room for discussion about their doubts.

*“If you want to read in the pregnant woman’s booklet*, *then you get the knowledge*. *But*, *about receiving information*… *no*! *Because prenatal consultations usually*… *they are like this*: *‘where are your exams*?*’*. *Or if you have not done any exams*, *you are asked to have them*. *And then*, *after the exams shown*, *if everything is fine*, *then you have to come once a month*. *Just*… *that’s pretty much it*, *right*? *To look at your face and say*: *‘are you all right*?*’ ‘All right*.*’ ‘All right then*. *Hugs’*.*”* [B3 –Alice]

*“Because she just wrote down my name and my SUS number on top*. *‘Go home*, *read your booklet*, *and you’ll understand*.*‴* [A1 –Vanessa]

*“I only received the booklet*, *and the only guidance they gave me was that ‘every time you come*, *you have to bring this booklet for us to sign*.*‴* [B4 –Lorena]

The Pregnant Woman’s Booklet is an instrument to monitor the evolution of pregnancy, in which health professionals must record all procedures and exams performed. In addition, it contains information on healthy pregnancy, baby development, guidelines on breastfeeding and women’s rights during pregnancy, among others [25]. The Booklet is a recognized and guaranteed right, and proved to be a robust information tool for pregnant women, who considered it informative and enlightening. They mentioned having easy access to it and being correctly oriented to always take it with them.

Although it is essential to value the Booklet and recommend reading it, this does not replace the need for the guidance given by professionals and the clarification of their doubts, using language appropriate to each context so that the information is understood. Such a posture favors embracement and strengthens the bond between the health team and the pregnant woman [39].

Some women reported using the internet and applications on mobile devices to circumvent the lack of guidance about the pregnancy-puerperal cycle.

*“There’s a lot that we don’t know*. *You don’t really know*. *Sometimes I stay at home*, *I do research*. *I have an app*, *I stay there not to do anything wrong*.*”* [A1 –Ana]

Apps have become popular among pregnant women who seek information about fetal health and development during pregnancy. However, there is a need for greater institutional involvement of health professionals in the development of these tools, as well as the provision of guidelines on the use for women, to avoid the spread of erroneous knowledge [40].

The lack of health education and guidance is reflected in women’s lack of knowledge about some of their rights. Although they recognize fundamental rights such as consultations, tests and vaccination, the reports express the users’ desire to learn more about the subject.

*“I*, *at least*, *don’t know my rights*. *Lack of guidance*, *right*? *We have no guidance*.*”* [B4 –Julia]

*“We don’t know [the rights]*. *You don’t have that knowledge*. *I don’t think it was ever informed*. *It is already my third pregnancy*, *and I don’t*… *I don’t know it*.*”* [A3 –Rosa]

Women recognized that knowledge about their rights would facilitate access to them.

*“We would know how to defend ourselves and put it there*, *right*? *Also*, *argue with the person when they think that we have no rights*. *Knowing that we have our rights*, *then we could say*: *‘oh*, *I’m sure my right is this*, *this and that and there’s no such a thing of you talking like that to me*!*’*.*”* [B4 –Amelia]

Health education promotes the autonomy and empowerment of individuals and social groups. There is evidence that community mobilization is an effective way to achieve this empowerment [7]. Therefore, it should be used as a strategy to improve individual health. The higher the level of education of the population, the greater the demand for social and health rights [7].

The insufficiency of activities aimed at health education in prenatal care can be attributed to the greater appreciation of objective issues, such as consultations and exams, indicating a biological focus on consultations [41]. This aspect is also emphasized in health care in quilombola communities in general. Cardoso et al. highlight that it tends to be localized and curative, focusing on the biological aspects of the health-disease process, healing, medicalizing, and being fragmented [19]. According to the authors, “this condition is aggravated further when associated with the rotation of professionals, precarious infrastructure for care and institutional racism. These factors are barriers that promote the increase of inequalities in health” [19].

Health professionals still find it challenging to adopt strategies that empower users, either because of their little involvement in activities or because of a lack of government support in this process [42]. Another explanation is that they adopt technical and educational strategies, such as delivering informative booklets, without producing meaning about the information, either through exchanges with professionals or other pregnant women [42]. Without the adoption of strategies that include the uniqueness of the population, which are not just women, but black women, poor, in specific territories, as in the case of quilombola communities, there is no possibility of empowerment. For this, it is also important to invest in the Permanent Education of the health teams from an intersectional perspective [43–45], attentive to the dimensions of gender, race and class, even more so when dealing with rural communities and more especially with black communities, such as the remaining quilombolas. In a society where sexism and racism are structural, different strategies are needed to face the processes that generate inequalities and iniquities. In order to guarantee access to quality care for black women from traditional communities, greater investments in Primary Health Care are essential, with the implementation of the National Policy for the Comprehensive Health of the Black Population (PNSIPN) [46].

Therefore, there is an urgent need to change the paradigm and model of health care for women, adopting strategies that consider their contexts and singularities. It includes promoting their actual participation in decisions and social engagement, such as home visits, listening workshops, and conversation circles [42]. A change in the power relationships established between health professionals and users is fundamental because even in the mentioned devices, relationships of subjection and devaluation of women’s experiences in their health and disease processes can be reproduced [42].

**Socioeconomic barriers.** In addition, two other difficulties pointed out, especially by users of municipality B, were the difficulty of moving to have access to the BHU and to the maternity. Two critical rights during prenatal care that, although not cited as rights, were claimed as necessary. Distance between the villages and lack of transport were identified as hindering the performance of consultations and exams during prenatal care, as well as causing difficulties in accessing the municipality’s maternity hospital, as they live in regions far from the health centers, sometimes more than an hour’s drive.

*“Then you need an ambulance*, *then call the municipality*, *and you don’t have it*, *if you don’t have the money to pay for a car*, *the situation is difficult”* [B3 –Alice]

Alice and Joana–the only women who declared themselves white–reported greater ease in accessing Rede Cegonha services. On the other hand, Ana was the only one that declared itself black and said it recognizes its rights. Such findings reinforce the persistence of race/color inequalities. In the rural municipality, the public transportation is only for the students. Other options for transport are limited. Communities who live far from the health centers usually use school transport to go to BHU in the early morning and return at the end of classes. The difficulty of accessing these communities, without public transportation and local health services, with great distances to be covered in the search for help, impacts the quality and accessibility of services [13, 19].

The lower prenatal coverage in the North and Northeast regions is associated more with barriers to access than with ignorance of pregnancy or personal problems of the pregnant woman [26]. Women living in rural areas have greater difficulty accessing public health services when compared to those in urban areas [47].

Many women reported using social benefits to getting around this situation, emphasizing the Bolsa Família Program.

*“Our condition here is very deficient*, *you know*? *At least the majority here receives only Bolsa Família*.*”* [B4 –Julia]

The Bolsa Família Program, created in 2003 as a strategy to fight poverty in Brazil, has great importance in the lives of the families that receive it since family income depends significantly on this benefit [48]. This program encourages families to seek preventive health care, with significant effects on pregnant women’s and children’s health [48]. The literature points out that the quilombola communities’ members, who generally carry out rural and/or subsistence work, still depend on this type of income transfer program [13].

All of these problems ─ distance and transport ─ are related to socioeconomic limitations, that are more severe in Brazilian afro-descendant populations, as the quilombolas. Racial vulnerability directly interferes with access to health services. In Brazil, women of brown/black race/color, who corresponded to most participants in this study, appear in worse socioeconomic conditions and prenatal care [49]. The black population has significant participation in the constitution of the Brazilian population, a majority presence among SUS users, and worse social and health indicators [50]. In addition to the historical exclusion marked by prejudices from society, this group is marked by the lack of specific actions to overcome this inequality [49] which justifies the need for specific policies to guarantee the rights and a better quality of life in these communities [51].

In 2009, the government established the PNSIPN [46]. This initiative sought to guarantee the right to health of this population by including themes such as racism and health of the black population in the training of health professionals, encouraging scientific production, recognizing popular health knowledge and practices, expanding access by the black population to health services, among others [46].

PNSIPN brought to the fore a discussion about intersections between gender and race as a specific point of action for the health of black women, since racial inequality interacts directly with gender inequality [43–45, 52]. Racism and sexism are structural factors that produce social hierarchies associated with health vulnerability, possibly associated with the worst social and health indicators of black women, contributing to the maintenance of high rates of early morbidity and mortality or preventable causes [50].

### Part 3. Vaccines in prenatal care: A consolidated right in Brazil?

Vaccination was presented as a positive point of prenatal care. Most interviewees recognized it as a right and reported that they had access to it.

*“[I received the vaccine] too much*! *My Pregnant Woman’s Booklet is full*. *Then we will take what is necessary for the child*, *it is more for the child that we take*, *right*? *And then it prevents many diseases*.*”* [B5 –Ligia]

*“I received all the vaccines here [at the BHU]*.*"* [A1 –Jessica]

Vaccination during pregnancy aims not only to protect the pregnant woman, but also the protection of the fetus. The Brazilian National Immunization Program (PNI), of the Ministry of Health, recommends the vaccination of pregnant women against diphtheria, tetanus, pertussis, hepatitis B and influenza [25, 53]. The PNI is a successful Brazilian public policy, which has contributed to a significant reduction in cases of vaccine-preventable diseases, such as measles, pertussis, polio, and diphtheria. PNI covers all life cycles and has different vaccination schedules for particular groups, such as pregnant women [53]. An example of a successful experience occurred in Alagoas, a state in the Northeast of the country. After an increase in pertussis cases in 2013, a reduction was observed after introducing dTpa (the vaccine against diphtheria, tetanus and pertussis) in the national calendar of the pregnant woman in 2014 [54].

Among users interviewed in the PMAQ-AB, 97% reported having updated the tetanus vaccine during prenatal care [30], consistent with the women’s report in our study. There has been much progress in vaccination coverage in Brazil and vaccination has been recognized by women in this study as a successful case of coverage and achievement of their right to health. However, the population’s search for vaccine in recent years has decreased, and the vaccination coverage has not reached the goals advocated by the WHO [55].

## Strengths, limitations, and political implications

To our knowledge, this is the first study to investigate the gaps that emerge from what the normative guidelines in relation to prenatal care establish and the actual experience of women, comparing two settings: one urban municipality and a rural municipality with a focus on quilombola communities. This comparison has implications for how healthcare, and the right to health, is studied and delivered. The findings of this study provide new insights for authorities and healthcare professionals. However, comparisons between the municipalities must be interpreted with caution, considering the singularities of each municipality.

Another strength relates to the spaces created for women to discuss their experiences during FG, opening new opportunities for the exchange of information and experiences during the discussions, as well as an enhanced awareness of health rights. In this way, issues addressed in this study align directly with the goal of reducing inequities in health, a significant goal that supports Brazil’s commitments to meet Sustainable Development Goals–particularly in regions still experiencing high maternal and infant mortality ratio.

Methodologically, this study was led by a commitment to give voice to women to talk about what they consider rights, and right to health in particular, and to discuss what they understand and experience as main barriers to access to quality healthcare. This is a novel and empowering way of creating knowledge about gaps and tensions in the delivery of healthcare and the right to health, a topic that is under-researched, understanding women’s views, particularly those in situation of poverty or marginalized is central. When women’s voices are muted or not heard, forms of gatekeeping and control in clinical decisions are made for them, disregarding their needs, wants and rights, and sometimes even without their understanding.

The participants interviewed were invited considering a timeframe of the 1000 days, that is from conception to when the child is two years old, so they talked about their pregnancy experiences, first hand and close to the occurrence of the events. This contributed to reducing recall bias. The questions presented in FG were opened and carefully worded to avoid influencing women’s narratives and constructions about their rights, leaving the participants free to talk about their experiences, positive or negative, avoiding direct interference in the answers by the facilitator.

On the other hand, there are two main limitations: i) Selection of women; and ii) External validation. Our research included more women from the municipality B, although this municipality was smaller than the municipality A. Even so, in both municipalities there was representation of the plurality of sociodemographic and contextual characteristics of these users of the health system, which provided diversity and richness of data and analysis. It is however important to acknowledge that because the sample selection was made by the CHW in the municipalities involved, it is possible that in the choice of women a potential selection bias may have included a sample with better care experiences during pregnancy and childbirth. Thus, it is possible to hypothesize that if the selection was made by the researchers, based on the medical records, the reported experiences could have been even more alarming. Nonetheless, almost all women reported negative experiences and there was consistency of responses.

There are several policy implications of this study. We identified the potential of the pregnant woman’s and child’s handbooks as instruments for disseminating rights in the first 1000 days. This information needs to be expanded and the distribution of booklets should be universal and perennial, as a priority for the management of the Brazilian health system. Unfortunately, however, the booklets are not being properly distributed, especially in places of greater social vulnerability. The policy of Permanent Education and Popular Education in the SUS needs to be strengthened, in order to qualify health professionals and contribute to the empowerment of the community, but it needs to consider aspects of the intersectionality of race, sex and income inequalities, otherwise it will remain ineffective. Public policies need to be continuously evaluated based on feedback from the target audience, allowing, for example, women to express their experiences, concerns and make suggestions. Universal health systems in contexts of scarce resources, such as in Brazil, need to implement creative and rational strategies to expand access to prenatal exams and strengthen the management of health care networks, making municipal borders more flexible to conform health regions that facilitate comprehensive care.

## Conclusions

Even in extreme poverty, Brazilian women have access to prenatal care in the public health system. However, there are still many challenges to the effective implementation of health actions and guaranteeing women’s rights. We offer an account of the experience of women regarding what they identified as barriers that threaten their right to health. These barriers particularly affect the right of women in rural communities, with lower socioeconomic levels and education, as well as brown and black women, who are already at greater health risk and inadequate prenatal care. As a consequence, one central challenge in the fulfilment of health needs and rights depend on closing what we identified as a performance gap between what the normative and legal frameworks encourage the health system to do and the lack of information that affect access, equality, respect and continuity of treatment amongst certain groups in society whose right to health are denied while their health risks increase.

Access barriers still hinder their effective guarantee of the identified rights. Adequate prenatal care, especially among women living in rural municipalities, prenatal dental care, delivery plan, and the link to maternity are not yet part of the reality of most pregnant women, and many of them do not even recognize them as rights. The intersectionality of race, gender and income inequalities is evidenced by inequity in healthcare access during prenatal care.

Women demonstrated a lack of knowledge about their rights. The lack of more guidance during prenatal care, both about caring for herself and the baby and her health rights, was evidenced. The Pregnant Woman’s Booklet then proved to be a great source of information for the women, as well as the internet and the use of mobile device applications. Also, the greater involvement of the health team in educational actions not restricted to biological aspects, seeking to empower women so that they can claim their rights and have greater access to quality health.
